# Point –of-care ultrasound diagnosis in seizures – A case report

**DOI:** 10.1186/2036-7902-4-S1-A15

**Published:** 2012-12-18

**Authors:** Monica Puticiu

**Affiliations:** 1 University Vasile Goldis, Arad, Romania

## Background

Systemic lupus erythematosus is a autoimmune disease that can affect any part of the body.

Seizure is a usual presentation at Emergency Department but systemic lupus erythematosus is a rare presentation.

We report a 21 years young women case present to Emergency Department with five seizures on the night. She was diagnosed two years ago with systemic lupus erythematosus and she was two weeks after caesarian surgery. She was confused and with right hemiparesis and Babinski sign. She had peripheral edema.

## Methods

We used point-of-care ultrasound to evaluate this young women.

We want to evaluate brain edema and we measured the optic nerve.

## Results

We find left and right optic nerve dimension 4.3 mm. We find bilateral pleural effusion, pericardial and abdominal effusion.

## Conclusion

Point-of-care ultrasound is a rapid and helpful tool in evaluation of the severity of systemic lupus erythematosus

The enlarged of optic sheath, 4.3 mm is not associate with brain edema on computed tomography.
